# Supplementary figures and images for: Comprehensive computational analysis of the SRK–SP11 molecular interaction underlying self-incompatibility in Brassicaceae using improved structure prediction for cysteine-rich proteins
Source: Comput Struct Biotechnol J. 2023 Oct 20;21:5228–39. doi: 10.1016/j.csbj.2023.10.026 (PMC10624595; doi:10.1016/j.csbj.2023.10.026)

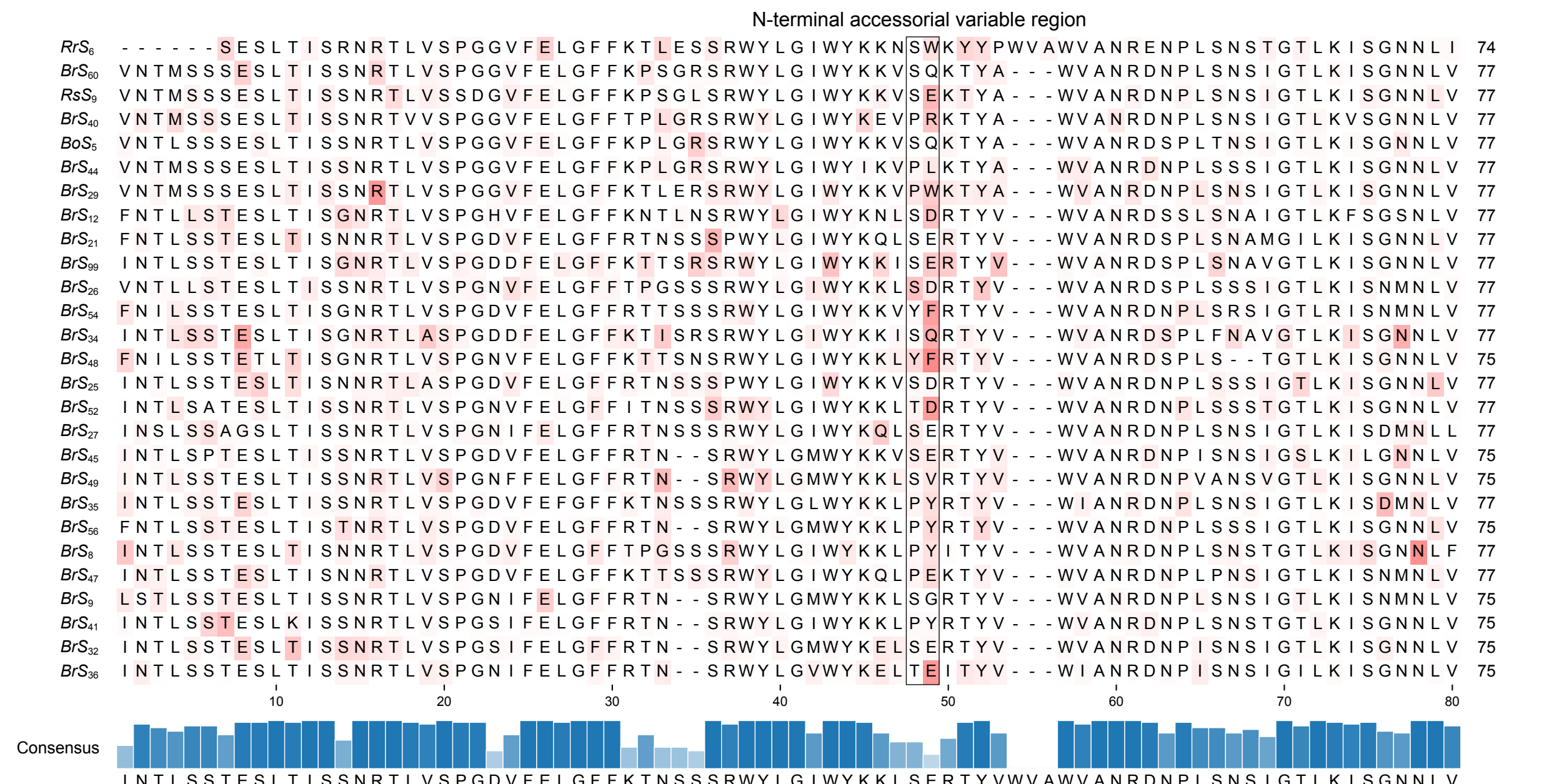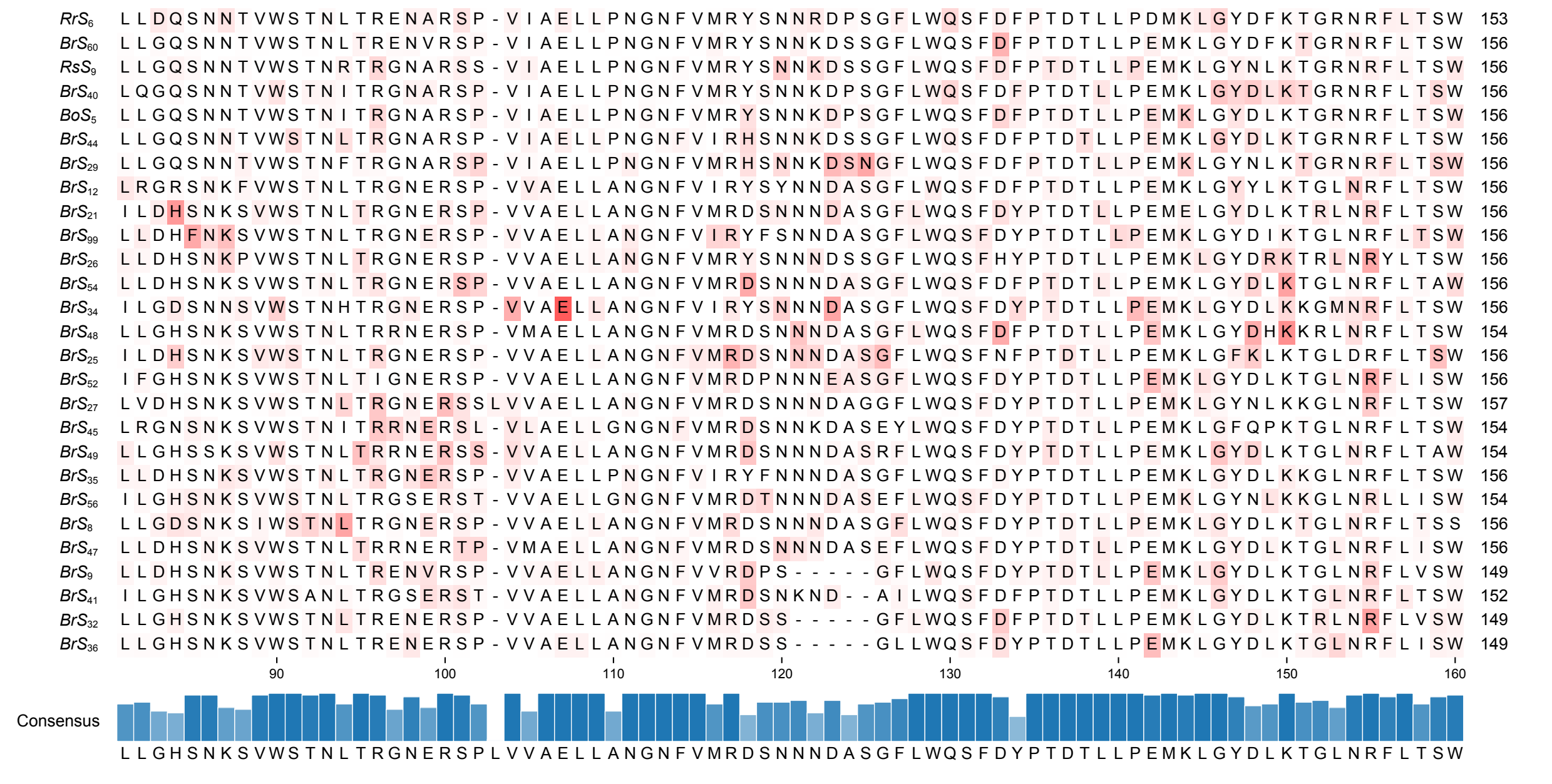

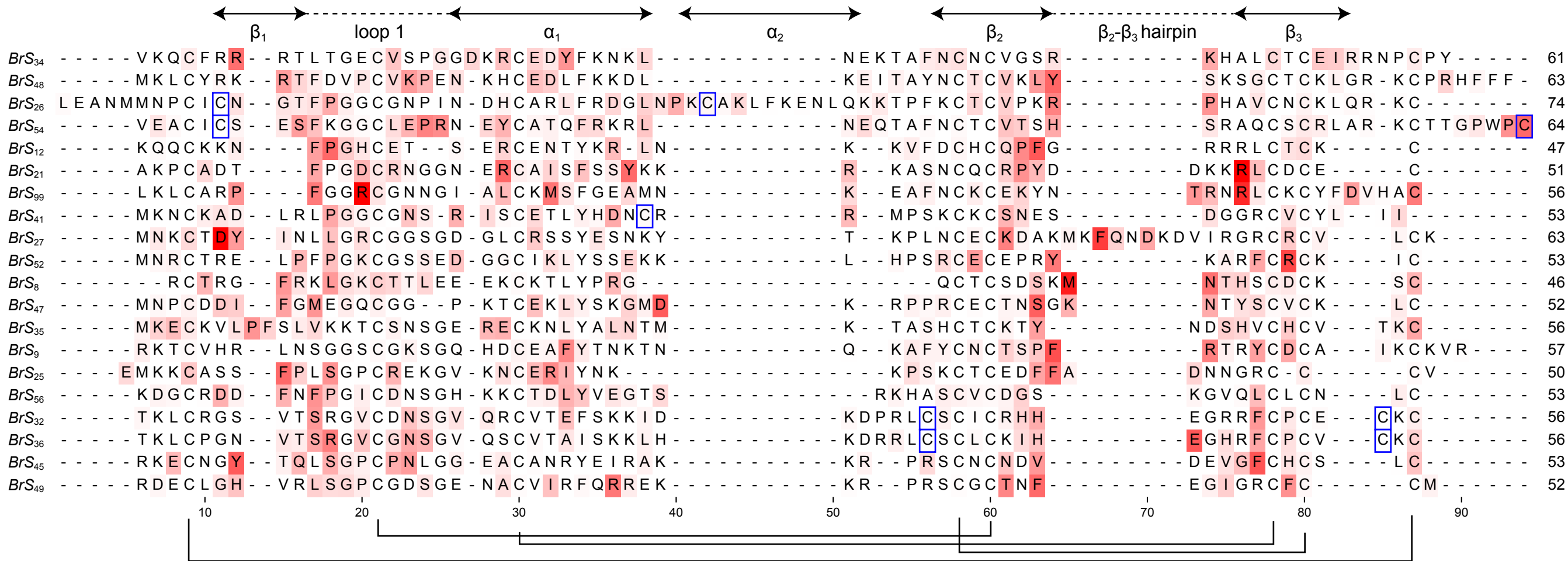



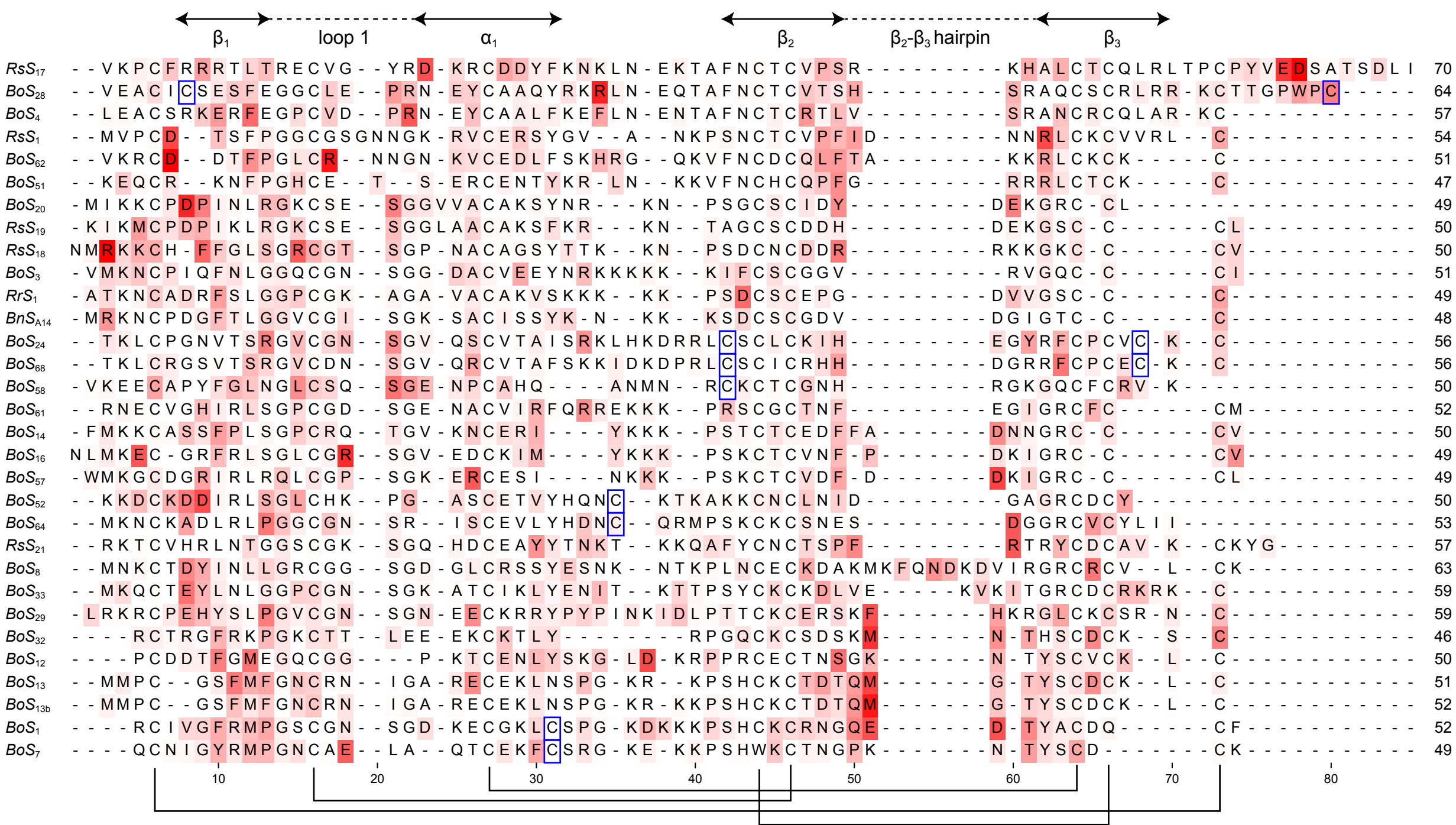

A

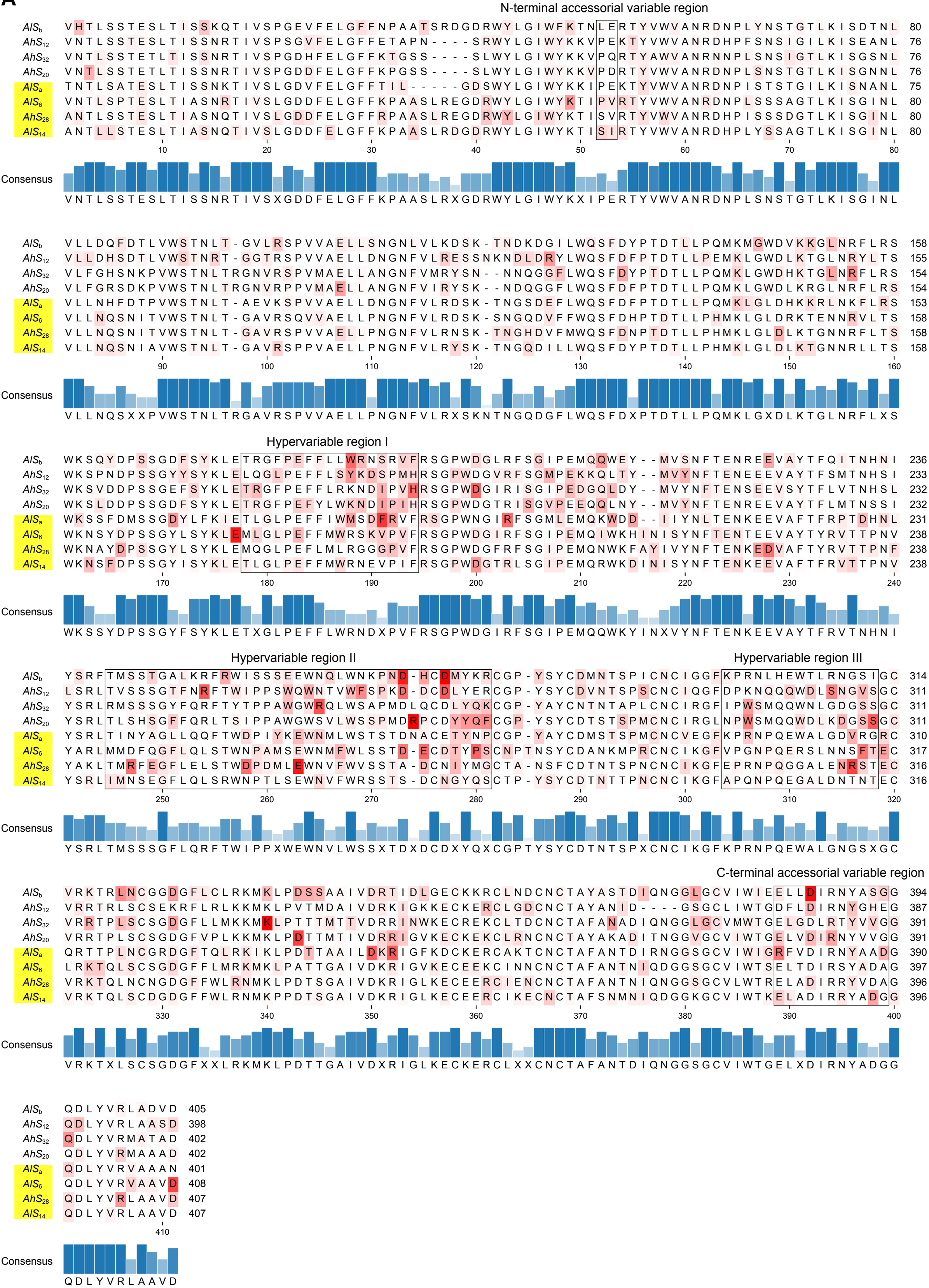

**B**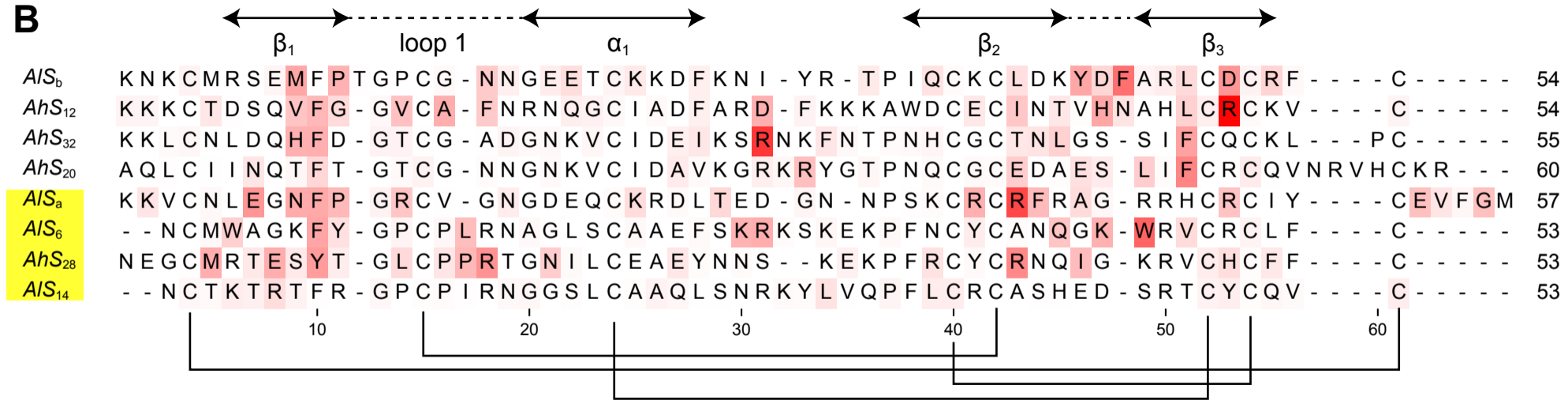

Supplement: Supplementary file 3 — Supplementary material [file mmc3.pdf]
