# Supplementary material for: Comprehensive computational analysis of the SRK–SP11 molecular interaction underlying self-incompatibility in Brassicaceae using improved structure prediction for cysteine-rich proteins
Source: Comput Struct Biotechnol J. 2023 Oct 20;21:5228–39. doi: 10.1016/j.csbj.2023.10.026 (PMC10624595; doi:10.1016/j.csbj.2023.10.026)
Supplement: Supplementary file 5 — Supplementary material [file mmc5.pdf]

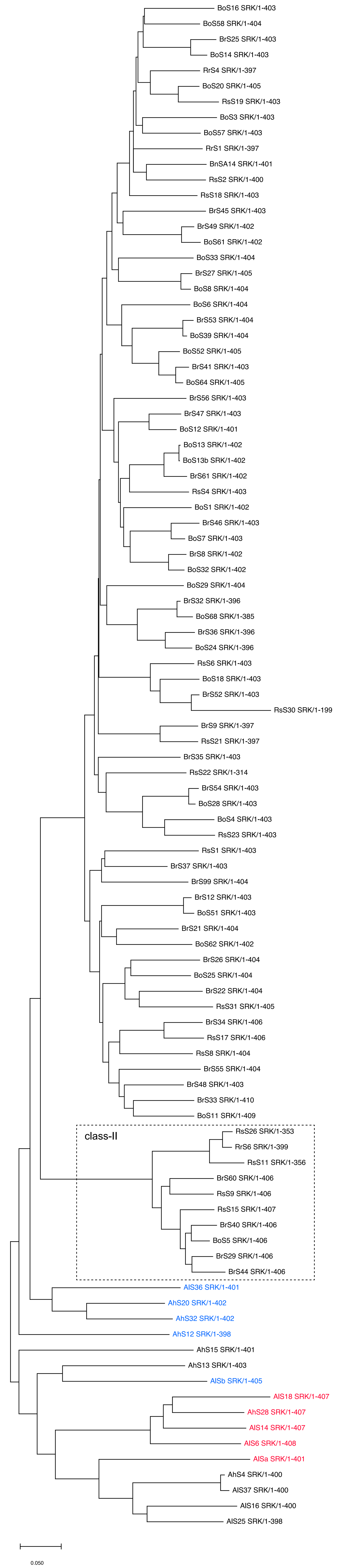

*Haplotypes of *Ah* and *Al* showing a similar binding mode to the crystal structure of *Br* S_8 or S_9 are colored in blue (Figure 5B) , and those with the different binding mode (Figure 5A) are in red.
